# The American Society in London

**Published:** 1920-11-27

**Authors:** 


### The American Society in London.

As this issue appears the Thanksgiving Day Dinner of the American Society in London is being held at the Hotel Cecil. The feature of the function is the honouring of British women who are pre-eminent in every sphere and station of life, and among those who will be present are:—Dame Margaret Lloyd George, G.B.E.; the Lady Ampthill, C.I., G.B.E., J.P., Chairman of Women V.A.D. Committee; Dame Katherine Furse, G.B.E., R.R.C., late Director, Women's Royal Naval Service; Miss Beadsmore-Smith, R.R.C., Matron-in-Chief, Q.A.I.M.N.S.; Miss Lloyd-Still, C.B.E., R.R.C., Matron and Superintendent, Nightingale Training School, St. Thomas's Hospital; Dame Maud McCarthy, G.B.E., R.R.C., Matron-in-Chief Territorial Force Nursing Service; Mrs. Mary Scharlieb, D.B.E., J.P., M.D., President London (R.F.H.) School of Medicine for Women; Miss Aldrich-Blake, M.S.; Lady Barrett, C.B.E., M.D., M.S., President London Association of Medical Women; Professor Winifred Cullis, O.B.E., D.Sc.; Miss Janet M. Campbell, M.D., Senior Woman Medical Officer, Ministry of Health, and Miss Margaret McMillan, C.B.E., Founder of School Clinics. Nearly all the speeches will be made by the lady guests, the only male speakers being the Chairman of the Society and the American Ambassador. Four hundred guests are expected, and the function is unique in the annals of public dinners.

